# Antigenotoxic Effect of *Trametes* spp. Extracts against DNA Damage on Human Peripheral White Blood Cells

**DOI:** 10.1155/2015/146378

**Published:** 2015-07-14

**Authors:** Aleksandar Knežević, Lada Živković, Mirjana Stajić, Jelena Vukojević, Ivan Milovanović, Biljana Spremo-Potparević

**Affiliations:** ^1^University of Belgrade, Faculty of Biology, Takovska 43, 11000 Belgrade, Serbia; ^2^University of Belgrade, Faculty of Pharmacy, Vojvode Stepe 450, 11000 Belgrade, Serbia

## Abstract

*Trametes* species have been used for thousands of years in traditional and conventional medicine for the treatment of various types of diseases. The goal was to evaluate possible antigenotoxic effects of mycelium and basidiocarp extracts of selected *Trametes* species and to assess dependence on their antioxidant potential. *Trametes versicolor, T. hirsuta*, and *T. gibbosa* were the species studied. Antigenotoxic potentials of extracts were assessed on human peripheral white blood cells with basidiocarp and mycelium extracts of the species. The alkaline comet test was used for detection of DNA strand breaks and alkali-labile sites, as well as the extent of DNA migration. DPPH assay was used to estimate antioxidative properties of extracts. Fruiting body extracts of *T. versicolor* and *T. gibbosa* as well as *T. hirsuta* extracts, except that at 20.0 mg/mL, were not genotoxic agents. *T. versicolor* extract had at 5.0 mg/mL the greatest antigenotoxic effect in both pre- and posttreatment of leukocytes. The mycelium extracts of the three species had no genotoxic activity and significant antigenotoxic effect against H_2_O_2_-induced DNA damage, both in pre- and posttreatment. The results suggest that extracts of these three species could be considered as strong antigenotoxic agents able to stimulate genoprotective response of cells.

## 1. Introduction 

Mushrooms have long been used as a food but equally in traditional medicine of both the western and eastern worlds [1]. Even though numerous mushrooms are recognized as healthy food [2, 3], their great pharmacological potential is still underutilized [4]. Nearly 60* Trametes* species are known to inhabit the world but just a few of them are screened for their medicinal properties [5].* Trametes versicolor* (L.:Fr.) Lloyd is the most famous medicinal species from the genus. This species, whose folk names are Turkey Tail in western cultures, Yun-Zhi (cloud-like mushroom) in China, or Kawaratake (mushroom by the river bank) in Japan, has been used for thousands of years in traditional medicine, particularly in Asia [6–8]. According to the Compendium of Chinese Materia Medica, written during the Ming Dynasty, more than 120 strains of* T. versicolor* have been recorded and in traditional Chinese medicinal practice this mushroom is considered useful for removing toxins, strengthening, energy increasing, improvement of liver and spleen function, and enhancing of the immune response, especially when it is dried, ground, and prepared into tea [7, 9–11]. All those properties were considered very useful in folk medicine for chronic use of* Trametes* spp. preparations [10]. In conventional medicine the species is used mainly for the treatment of various types of cancers, but also for chronic hepatitis, rheumatoid arthritis, and infections of the respiratory, urinary, and digestive tracts, which was confirmed by numerous studies [6, 10–14]. Additionally, strong antiviral effects of some polysaccharopeptides isolated from* T. versicolor* and significant antioxidant activity of* Trametes* spp. fruiting body extracts have been reported [15–17]. These effects are mainly based on production of the polysaccharide krestin (PSK) and various polysaccharide-peptide complexes, compounds which reduce cancer metastases and stimulate the production of interleukin-1 in human cells [18–20].

The abundant presence of free radicals in the environment is associated with the appearance of oxidative stress which is a basis of aging and the initiation and progress of various diseases and disorders from which a large part of the world's population suffers and dies [21]. DNA is more sensitive to oxidative damage than other macromolecules. DNA damage, such as strand breaks, could be induced by various agents among which H_2_O_2_ produces a genotoxic effect. It is known that those damages can affect the immune response not only in inflammatory diseases but also in cancers [22, 23]. The comet test is a well-established and effective test of high sensitivity that has been used for examining DNA damage and can be applied to assess the genotoxic and protective potential of several natural products [24–26].

A genoprotective activity of mushroom extracts based on the reduction of oxidative damages of DNA can also play a significant role in prevention and treatment of several mentioned diseases and disorders but very few studies until nowadays considered it as a possible tool of action in different therapies [27, 28]. Therefore the goal of the study was to evaluate antigenotoxic effects of mycelium and basidiocarp extracts of selected* Trametes* species on human peripheral white blood cells and to assess dependence on their antioxidant potential.

## 2. Materials and Methods

### 2.1. Organisms and Cultivation Conditions

Cultures of* Trametes versicolor* BEOFB 321,* T. hirsuta* BEOFB 301, and* T. gibbosa* BEOFB 310 were isolated from fruiting bodies collected from Serbia and maintained on Malt agar medium in the culture collection of the Institute of Botany, Faculty of Biology, University of Belgrade (BEOFB).

The inoculum was prepared by inoculation of 100.0 mL of synthetic medium (glucose, 10.0 g L^−1^; NH_4_NO_3_, 2.0 g L^−1^; K_2_HPO_4_, 1.0 g L^−1^; NaH_2_PO_4_ × H_2_O, 0.4 g L^−1^; MgSO_4_ × 7H_2_O, 0.5 g L^−1^; yeast extract, 2.0 g L^−1^; pH 6.5) with 25 mycelial disks (Ø 0.5 cm, from 7-day-old culture from malt agar) in 250 mL flasks and incubation on a rotary shaker at 100 rpm, at room temperature (22 ± 2°C) for 7 d. The resultant biomass was washed and homogenized with 100.0 mL of sterile distilled water (dH_2_O) in a laboratory blender. Homogenized biomass (30.0 mL) was used for inoculation of 500.0 mL modified synthetic medium (with glucose present at 65.0 g L^−1^). Submerged cultivation was carried out in 1000 mL flasks at room temperature on a rotary shaker for 21 d. The obtained biomass was filtered, washed 3 times with dH_2_O on a magnetic stirrer, and dried at 50°C to constant weight.

### 2.2. Preparation of the Fungal Extracts

Dried fruiting body and mycelium (3.0 g) were extracted by stirring with 90.0 mL of 96% ethanol at 30°C for 72 h. The resulting extracts were centrifuged (20°C, 3000 rpm, 15 min) and supernatants were filtered through Whatman number 4 filter paper, concentrated under reduced pressure in a rotary evaporator (BÜCHI R-114, Switzerland) at 40°C to dryness, and redissolved in 96% ethanol for antioxidant assay [29] or water for antigenotoxic assay [30] to an initial concentration of 20.0 mg mL^−1^. The extraction yield was expressed as percentage on a dry weight basis.

### 2.3. Genoprotective Activity

#### 2.3.1. Subjects

Heparinized whole blood samples were obtained by venipuncture from three healthy donors aged under 25. Participants of the study were nonsmokers and nonalcoholics, not receiving any therapy or medications and not taking dietary supplements.

#### 2.3.2. Study Design

Genotoxicity of all extracts and concentrations (20.0, 10.0, 5.0, 2.5, 1.25, 0.625, and 0.312 mg mL^−1^) was studied by treatment of human peripheral white blood cells at 37°C for 30 min with the aim of evaluating DNA damage. Normally, white blood cells are used, because they are obtained in a relatively noninvasive way, do not require tissue disaggregation, and behave well in the comet assay [31]. Treatment with phosphate buffered saline (PBS) at 37°C for 30 min was used as a positive control and treatment with 25.0 *μ*M H_2_O_2_ on ice for 15 min as a negative control.

Two independent protocols were used to assess the antigenotoxic potential of extracts, using pretreatment and posttreatment with the extracts. In pretreatment, the cells were incubated with extracts at 37°C for 30 min, then washed with PBS, and exposed to H_2_O_2_ for 15 min. In posttreatment, the cells were treated with H_2_O_2_ on ice for 15 min, rinsed with PBS, and subsequently treated with the seven extract concentrations at 37°C for 30 min. After each treatment, the cells were washed with PBS. Incubation with PBS at 37°C for 30 min was the negative control and treatment with 25.0 *μ*M H_2_O_2_ on ice for 15 min represented the positive control.

Three replicates were performed for each experiment and 100 nuclei were analyzed for each.

#### 2.3.3. The Single Cell Gel Electrophoresis Assay

The comet assay was performed as described by Singh et al. [32]. The alkaline comet test is able to detect DNA strand breaks and alkali-labile sites, and the extent of DNA migration indicates the degree of DNA damage in cells.

**Figure 1:**
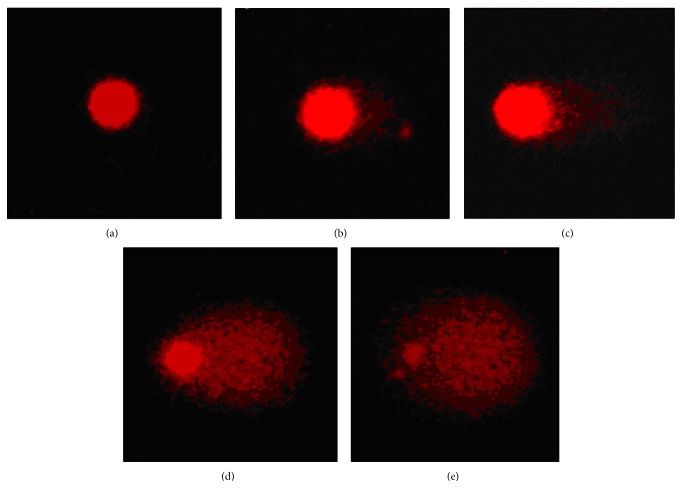
Categorisation of DNA damage corresponding to the amount of DNA in the tail.

Whole blood samples (6.0 *μ*L) were suspended in 0.67% low-melting-point (LMP) agarose (Sigma-Aldrich, St. Louis, MO) and pipetted onto superfrosted glass microscope slides precoated with a layer of 1% normal-melting-point agarose (Sigma-Aldrich, St. Louis, MO), spread using a cover slip, and maintained on ice for 5 min to solidify. After gently removing the cover slips, the cell suspensions on slides were treated with the extracts and H_2_O_2_ as described above. Following the treatments, all slides were covered with the third layer of 0.5% LMP agarose and again was allowed to solidify on ice for 5 min. After removal of the cover slips, the slides were placed in cold lysing solution (2.5 M NaCl, 100 mM EDTA, 10 mM Tris, 1% Triton X100, and 10% dimethyl sulfoxide, pH 10.0 adjusted with NaOH) at 4°C overnight and afterwards subjected to electrophoresis and staining with ethidium bromide [32]. The comets were observed and analyzed using an Olympus ×50 microscope (Olympus Optical Co., Gmbh Hamburg, Germany), equipped with a device for recording fluorescence at 100x magnification. Evaluation of DNA damage was performed as described by Anderson et al. [24]. Namely, cells were graded by eye into five categories corresponding to the following amounts of DNA in the tail: (A) no damage, <5%; (B) low level damage, 5–20%; (C) medium level damage, 20–40%; (D) high level damage, 40–95%; (E) total damage, >95% (Figure 1). Analysis was performed on 100 randomly selected cells per subject (50 cells from each of 2 replicate slides). To obtain semiquantitative analysis of data, DNA damage was characterized as DNA migration over 5% (B + C + D + E comet classes).

### 2.4. Antioxidant Activity

#### 2.4.1. DPPH^•^ Assay

Antioxidant activity was defined by measuring bleaching of the purple-colored methanol solution of stable 1,1-diphenyl-2-picrylhydrazyl radical (DPPH^•^) [33]. Scavenging effects were measured spectrophotometrically (CECIL CE 2501) at 517 nm and calculated using the equation:

(1) DPPH•  scavenging  effect  %=A0−AsampleA0−1×100,

where *A*_0_ is absorbance of the negative control (reaction mixture without extract) and *A*_sample_ is absorbance of reaction mixture.

Extract concentration (mg extract/mL) providing 50% of DPPH^•^ reduction (EC_50_) was obtained by interpolation from linear regression analysis. All the measurements were carried out in triplicate for statistical analysis. Commercial antioxidant, butylated hydroxyanisole (BHA), in a concentration range of 20.0 mg mL^−1^–0.02 mg mL^−1^, was used as a positive control.

#### 2.4.2. Determination of Total Phenol Content

Total phenol compounds in the mycelial extracts were estimated with Folin-Ciocalteu reagent according to the method of Singleton and Rossi [34], using gallic acid as a standard. The concentration was determined as *μ*g of gallic acid equivalents (GAE) per mg of dry extract, using an equation that was obtained from a standard gallic acid graph as

(2) Absorbance=0.012×total  phenolsμg  of  gallic  acid−0.029R2=0.999.

#### 2.4.3. Determination of Total Flavonoid Content

Total flavonoid content was determined by the methods of Park et al. [35] using quercetin as the standard. The amount was expressed as *μ*g of quercetin equivalents (QE) per mg of dry extract, using an equation obtained from a standard quercetin hydrate graph as

(3) Absorbance=0.011×total  flavonoidsμg  of  quercetin  hydrate+0.080R2=1.0.

### 2.5. Statistical Analysis

The results were expressed as the mean ± standard error of data obtained from three parallel measurements. One-way analysis of variance (ANOVA) was performed using STATISTIKA software, version 5.0 (StatSoft Inc.) to test any significant differences. *P* values less than 0.01 were considered statistically significant. The statistical analysis of data from the comet assay was performed by *χ* ^2^ test using Statgraph 4.2 software. To perform *χ* ^2^ test, results from the three experiments were pooled and we evaluated total number of cells with DNA damages. A difference at *P* < 0.05 was considered statistically significant.

## 3. Results and Discussion

### 3.1. Extraction Yield

Extraction yields of mycelium biomass for all three species were significantly higher compared with the fruiting body (*P* < 0.01).* T. gibbosa* had the highest extraction yield from dried mycelium biomass (34.6%) and the lowest yield from dried fruiting bodies (2.2%). The highest fruiting body extraction yield of 6.67% was found in* T. versicolor*, whose mycelium extraction yield was 8.0%. Yields in* T. hirsuta* were 12.0% (for mycelium) and 2.85% (for fruiting body). Differences in extraction efficiency among the species, for both mycelium and fruiting body, were statistically significant (*P* < 0.01).

Previous reports showed the dependence of biomass extractability on species, strain, and solvent [36–38]. Thus, Ren et al. [37] found that extraction yields of* T. gibbosa* basidiocarp were 1.22% for petroleum ether extract, 6.44% for ethyl acetate, and 9.2% for methanol extracts. Methanol was also a good solvent for* T. versicolor* basidiocarp whose yield ranged between 4.1% and 9.16% [36, 38]. Based on our results, it can be concluded that alcohols are the best solvents, but ethanol is weaker than methanol.

### 3.2. Genoprotective Activity

As all blood donors were of good health and similar age and under no medication, the statistical analysis showed no clear differences in their responses to extracts. Therefore, results from the three experiments were pooled. Treatment of peripheral blood leukocytes with H_2_O_2_ caused a quick and powerful induction of single strand breaks in the nuclear DNA, which was visible in the comet assay as DNA migration.

**Figure 2:**
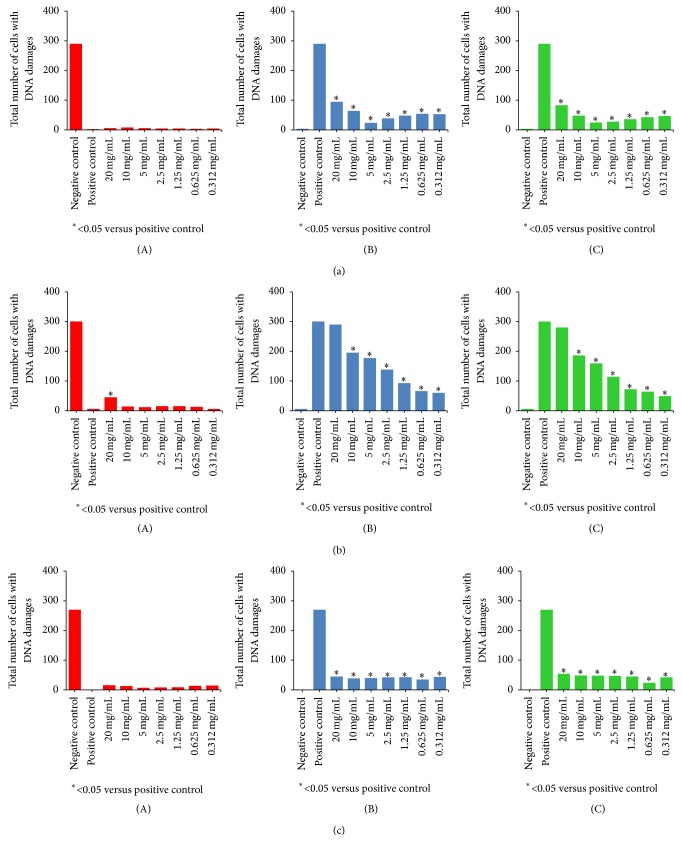
Effect of fruiting body extracts of (a)* Trametes versicolor,* (b)* T. hirsuta,* and (c)* T. gibbosa*: (A) genotoxic, (B) antigenotoxic, pretreatment, and (C) antigenotoxic, posttreatment. Three independent experiments with three replicates per experiment were done and evaluated by comet assay. 100 nuclei per each replicate were analyzed. Data represent total number of cells with DNA damage.

Our results demonstrated that* T. versicolor* fruiting body extracts from 0.312 to 20.0 mg mL^−1^ caused no significant increase in the total number of DNA-damaged cells compared with the positive control, which clearly shows that the tested extract was not a genotoxic agent (Figure 2(a) (A)). The distribution (value) of total DNA damage was also the same as in the positive control. On the other hand, these extracts showed protective effects against H_2_O_2_ both in pre- and posttreatment of leukocytes (Figure 2(a) (B, C)). The extract at 5.0 mg mL^−1^ had the greatest effect and at 20.0 mg mL^−1^ the lowest effect in both treatments. The value of total DNA damage statistically decreased compared with the positive control in all concentrations (*P* < 0.05).

*T. hirsuta* fruiting body extract at all concentrations except 20.0 mg mL^−1^ showed no genotoxic activity as the level of total DNA damage was not statistically higher than that in the positive control (Figure 2(b) (A)). However, at a concentration of 20.0 mg mL^−1^, the genotoxic effect and total DNA damage in cells were statistically different compared with the positive control. In pre- and posttreatments of leukocytes, the extract at all concentrations except the highest one exhibited a protective effect against H_2_O_2_-induced DNA damage, showing significant decrease of total DNA damage compared with the positive control (Figure 2(b) (B, C)). These treatments displayed a dose-dependent correlation, with the greatest protective effect at an extract concentration of 0.312 mg mL^−1^ while the concentration of 20 mg mL^−1^ showed no protection against comets induced by H_2_O_2_.

The absence of a genotoxic as well as significant antigenotoxic effect, that is, reduction of DNA damage induced by H_2_O_2_, in both pre- and posttreatment, was also noted for* T. gibbosa* fruiting body extract at the seven concentrations (Figure 2(c)). However, contrary to* T. hirsuta* extracts, a dose-dependent response was not observed in* T. gibbosa* basidiocarp extracts; namely, a gradual decrease of extract concentration did not correspond with a proportional reduction of H_2_O_2_-induced genotoxicity.

**Figure 3:**
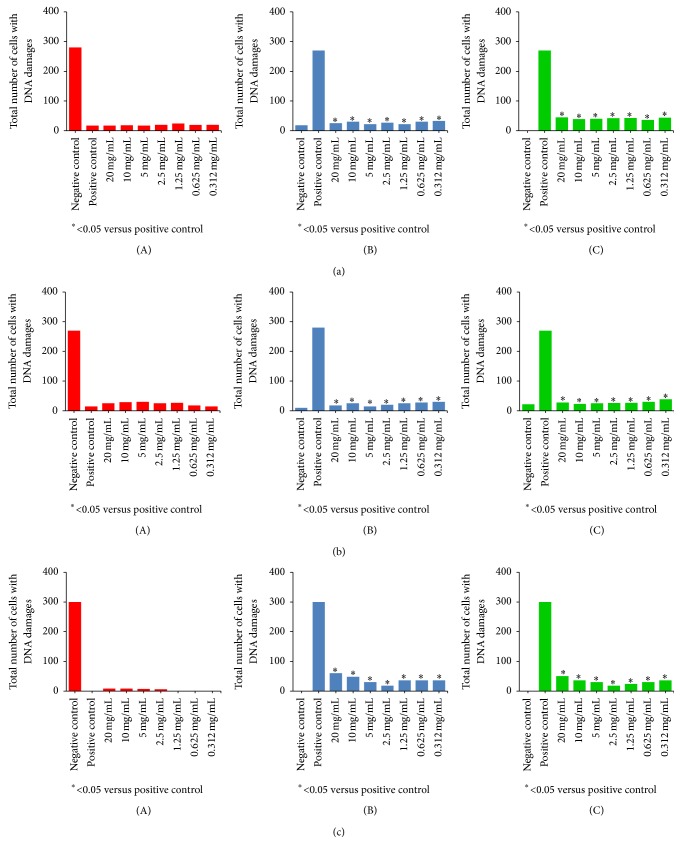
Effect of mycelium extracts of (a)* Trametes versicolor,* (b)* T. hirsuta,* and (c)* T. gibbosa*: (A) genotoxic, (B) antigenotoxic, pretreatment, and (C) antigenotoxic, posttreatment. Three independent experiments with three replicates per experiment were done and evaluated by comet assay. 100 nuclei per each replicate were analyzed. Data represent total number of cells with DNA damage.

The mycelium extracts of* T. versicolor*,* T. hirsuta,* and* T. gibbosa*, at all analyzed concentrations, had no genotoxic activity (Figures 3(a) (A), 3(b) (A), and 3(c) (A)). All mycelium extracts and concentrations showed a significant antigenotoxic effect against the H_2_O_2_-induced DNA damage, both in pre- and posttreatment, and these activities were not markedly different. In* T. versicolor*, a slightly lower activity was noted at the lowest extract concentration. In* T. hirsuta*, concentrations of 5.0, 2.5, and 20.0 mg mL^−1^ were more effective, while in* T. gibbosa* the greatest protective effect was observed at a concentration of 2.5 mg mL^−1^ and the lowest one at 20.0 mg mL^−1^ (Figures 3(a) (B, C), 3(b) (B, C), and 3(c) (B, C)).

Numerous mutagenic and carcinogenic compounds are present in different natural sources [39]. On the other hand, some natural compounds could be either prooxidants causing genotoxic and/or cytotoxic effects or antioxidants, depending on the concentration and duration of exposure [40–43]. Highly nutritional and medicinally valued mushroom species may have different* in vitro* and* in vivo* effects due to either their instability under digestion conditions or inability of absorption by the gastrointestinal tract [44]. Namely, activities obtained* in vitro* do not necessarily correspond to those found* in vivo*. It is also important to emphasize that genotoxic and antigenotoxic effects of mushroom extracts depend on species, concentration, and the assay used for their assessment [44–47]. Thus, our results demonstrated different capacities of the three* Trametes* species for decreasing H_2_O_2_-induced DNA damage; for example, the lowest activity was noted in fruiting body extract of* T. hirsuta*. A clear inverse dose-response relationship between the level of DNA damage and extract concentration was noted only in* T. hirsuta* basidiocarp extract. However, in* T. versicolor* and* T. gibbosa*, increasing the extract concentration above the optimal dose did not lead to any improvement in comet results which confirms results of Miyaji et al. [40]. These authors showed the absence of a dose-response relationship between* Lentinus edodes* extract concentrations and their antigenotoxic effect. It is important to mention that combined phenolic, flavonoid, and other ingredients in extracts should have greater potential than individual components of extracts, indicating the significance of coactions of all ingredients [48]. That finding could result in different trend of* Trametes* spp. antigenotoxic activity. Dependence of the genotoxic activity of the extract on the assay type was demonstrated by Morales et al. [47]; that is, they reported absence of mutagenic effect of basidiocarp extracts of* Lactarius deliciosus*,* Boletus luteus, Agaricus bisporus,* and* Pleurotus ostreatus* on mammalian cells using the Ames Salmonella/microsome test. However, a weak activity of* P. ostreatus* extract was obtained using the CHO/HPRT assay.

The underlying mechanisms of the antigenotoxic effect of mushroom extracts are still not completely known. Protective effects of the extracts seem to be based on more than one mechanism of action, which is not uncommon for mushrooms according to Gebhart [49]. The antigenotoxicity mechanisms could be evaluated by applications of pre- and posttreatments, that is, diverse combinations of extracts and H_2_O_2_. Our positive results in both treatments indicate that extracts have protective effects at both the prevention and intervention levels and may act as desmutagens and bioantimutagens, also demonstrated by previous studies [50–52]. Efficiency of pretreatment, noted in the present study, could be explained by increasing the antioxidant capacity of cells, that is, stimulating the synthesis and activity of antioxidant enzymes during the induction of oxidative stress [53]. The positive effect of posttreatment could be the result of synergistic action of interventional activities* via* free radical scavenging and stimulation of antioxidant enzymes, as well as excitation of DNA repair, as suggested by Chiaramonte et al. [54]. As these authors reported significant DNA damage repair after 30–60 min of exposure to an oxidative agent, it could be concluded that DNA repair played a less significant role in protection against H_2_O_2_ since posttreatment conditions considered up to 30 min of incubation. Therefore, the genoprotective activity of the* Trametes* spp. extracts is probably based on antioxidant actions. On the other hand, it is known that eukaryotic organisms have evolved a signaling pathway, called the DNA damage response, to protect against genomic insults. Gasser and Raulet [22] demonstrated that the DNA damage response alerts the immune system by inducing expression of cell surface ligands for the activating immune receptor NKG2D, which is expressed by natural killer cells (NK cells) and some T cells. Therefore, the genoprotective activity of* Trametes* spp. in the cells exposed to genotoxic agents could modulate DNA damage response and function as a barrier in early tumorigenesis. The further researches should include analysis of superoxide dismutase and catalase levels in lymphocytes treated with* Trametes* spp. extracts, both in pre- and posttreatment with H_2_O_2_, in order to confirm assumption that enhancement of antioxidant capacity in cells is induced by those extracts.

### 3.3. Antioxidant Activity

The tested ethanol extracts were good antioxidants but their activity depended on species. Fruiting body extracts showed significantly higher scavenging effects than mycelium extracts (*P* < 0.01). The highest DPPH radical scavenging activity was detected in* T. versicolor* extracts, both fruiting body and mycelium (63.5% and 59.4%, resp.) which was confirmed by EC_50_ values (15.22 mg mL^−1^ and 16.18 mg mL^−1^, resp.). A slightly lower level of activity was found for* T. hirsuta* extracts (59.0% for basidiocarps and 46.8% for mycelium), whose concentrations of 17.06 mg mL^−1^ and 21.81 mg mL^−1^, respectively, provided a 50% reduction of radicals.* T. gibbosa* was the species with the lowest DPPH^•^ scavenging potential, especially of mycelium extracts (39.7%) with EC_50_ value of 26.15 mg mL^−1^. However, the radical scavenging ability of the fruiting body extract was not significantly lower in comparison with the other two species (53.7% and EC_50_ of 18.13 mg mL^−1^). The DPPH^•^ scavenging activity of synthetic antioxidant BHA was 94.28%, and a concentration of 0.10 mg mL^−1^ provided DPPH^•^ reduction of 50%.

Total phenol contents in fruiting body and mycelium extracts of* Trametes* species were significantly different (*P* < 0.01) (Table 1). Generally, phenol contents in fruiting body extracts were higher than in mycelium extracts.

Both* T. versicolor* basidiocarp and* T. versicolor* mycelium extracts were the richest with phenols and flavonoids, while the lowest concentrations were measured in* T. gibbosa* extracts. According to the phenol and flavonoid concentrations, extracts of* T. hirsuta* came between the other two species extracts (Table 1). The degree of correlation between the DPPH^•^ scavenging activity of the extracts and phenol and flavonoid contents was high with *R* ^2^ for fruiting bodies of 0.98 and 0.99, respectively, and for mycelium of 0.97 and 0.99, respectively.

Previous studies have also indicated the antioxidant potential of* Trametes* species [17, 55, 56]. Thus, Kamiyama et al. [56] demonstrated that an extract concentration of even 0.5 mg mL^−1^ scavenged nearly 50% of DPPH^•^ depending on the solvent, while Johnsy and Kaviyarasana [55] noted reduction of even 91.5% radicals by a methanol extract of* T. gibbosa* basidiocarps at a concentration of 1.0 mg mL^−1^. Ethanol extracts tested in our study had slightly lower capacities, but higher than ethanol* T. hirsuta* fruiting body extracts analyzed by Sheikh et al. [17].

According to Mau et al. [57] and Palacios et al. [58], phenolic compounds play a key role in antioxidative activity. These compounds are very abundant and important constituents of mushroom fruiting bodies and mycelia. Their ability is based on the presence of hydroxyl groups acting as reducing agents, metal chelators, singlet oxygen quenchers, and hydrogen donors [59]. However, in some cases their activity could not be attributed to the total phenol content in extracts, which is confirmed by comparison of our results with those of Johnsy and Kaviyarasana [55]. Namely, 91.5% of DPPH^•^ was reduced by* T. gibbosa* basidiocarp extract containing 23.8 *μ*g GAE mg^−1^ extract, while an extract of strain BEOFB 310 with a phenol concentration of 20.07 *μ*g GAE mg^−1^ extract scavenged only 63.5% radicals. However, the concentration of flavonoids in the Serbian* T. gibbosa* strain was significantly higher compared with the strain tested by Johnsy and Kaviyarasana [55] (7.63 *μ*g QE mg^−1^ of extract and 0.59 *μ*g QE mg^−1^ of extract, resp.), and this could be explained by the various polarities of solvents as well as different strain capacity for flavonoid synthesis [60].

## 4. Conclusion

The study was the first attempt to assess the DNA protective activity of* T. versicolor*,* T. hirsuta,* and* T. gibbosa* extracts and determines whether this was based on their antioxidant potential. The results suggest that extracts of these three species could be considered as strong antigenotoxic agents able to stimulate genoprotective response of cells contributing to enhanced immune function, toxin removal, and strengthening, which refers to the traditional use. However, further investigations are necessary to reveal specific carriers of the antigenotoxic activity and the mode of DNA protection from oxidative damage.

## Figures and Tables

**Table 1:** Total phenol and flavonoid content in ethanolic extracts of selected *Trametes* species.

Tested species	Extract	Total phenol content	Total flavonoid content
		(*µ*g GAE/mg of dried extract)	(*µ*g QE/mg of dried extract)
*Trametes gibbosa *	Basidiocarp	20.07 ± 1.24	7.63 ± 0.08
	Mycelium	12.08 ± 0.87	1.76 ± 0.03

*Trametes hirsuta *	Basidiocarp	21.53 ± 2.36	8.28 ± 0.05
	Mycelium	14.27 ± 0.92	2.21 ± 0.02

*Trametes versicolor *	Basidiocarp	24.80 ± 0.42	10.79 ± 0.09
	Mycelium	18.06 ± 0.33	4.16 ± 0.02
